# Why are self-medication opportunities limited in Austria? An interview study and comparison with other countries

**DOI:** 10.1371/journal.pone.0245504

**Published:** 2021-01-25

**Authors:** Natalie J. Gauld, Christoph A. Baumgärtel, Stephen A. Buetow

**Affiliations:** 1 School of Pharmacy, The University of Auckland, Auckland, New Zealand; 2 Austrian Federal Office for Safety in Health Care, BASG/Austrian Agency for Health and Food Safety, AGES, Vienna, Austria; 3 Department of General Practice and Primary Health Care, The University of Auckland, Auckland, New Zealand; BCS Health & Care Scotland, UNITED KINGDOM

## Abstract

**Background:**

Austria has high health resource use compared to similar countries. Reclassifying (switching) medicines from prescription to non-prescription can reduce pressure on health resources and aid timely access to medicines. Since Austria is less progressive in this area than many other countries, this research aimed to elucidate enablers and barriers to it reclassifying medicines and make recommendations for change in the context of similar research conducted elsewhere.

**Methods:**

Qualitative research using a heuristic approach was conducted in Austria in 2018. Informed by their own “insider” and “outsider” knowledge, the authors identified themes from personal interviews with 24 participants, including reclassification committee members, government officials and stakeholders, before comparing these themes with earlier research findings.

**Results:**

Significant barriers to reclassification included committee conservatism, minimal political support, medical negativity and few company applications. Insufficient transparency about committee decisions, expectations of adverse committee decisions and a limited market discouraged company applications. Austria’s ‘social partnership’ arrangement and consensus decision making aided a conservative approach, but the regulator and an alternative non-committee switch process were enabling. Pharmacy showed mixed interest in reclassification. Suggested improvements include increasing transparency, committee composition changes, encouraging a more evidence-based approach by the committee, more pharmacy undergraduate clinical training, and companies using scientific advisory meetings and submitting high quality applications.

**Conclusion:**

Removing barriers to reclassification would facilitate non-prescription availability of medicines and encourage self-care, and could reduce pressure on healthcare resources.

## Introduction

Increasing self-care offers a possible way to contain health care costs in Europe [1], a challenge which is likely to become even more important in managing the economic consequences of a Covid-19-related recession [2]. To facilitate self-care, reclassification (or switching) of medicines from prescription to non-prescription has been taking place internationally for decades. This development has taken place despite variability in reclassification between countries [3]. The widened access to medicines from reclassification could benefit individuals, health funders and society. It could save time for patients, save health resources, empower patients, and enable more timely treatment of conditions [1, 4–7]. It could also help to address undertreatment of conditions, aid public health initiatives (e.g. smoking cessation and vaccination) and increase the time available to doctors to attend to serious medical needs. However, reclassification also poses risks.

Potential downsides of reclassification include adverse events, misuse or abuse of medicines, reduced opportunistic screening, and delayed diagnosis of important conditions [4, 6]. Strategies such as restrictions on availability (e.g. only through a pharmacy), label-warnings, pack size limitations, training for pharmacy staff, and screening tools have been developed to address potential risks. International comparative research can provide valuable insights to balance benefits and risks [8].

In a 16 country comparison, Austria was one of the most restrictive nations in the range of medicines requiring a prescription [5]. Nasal corticosteroids for allergy, triptans for migraine, antibiotic eye preparations for conjunctivitis, and dermal hydrocortisone for dermatitis exemplify medicines found to require a prescription in Austria but which have been reclassified elsewhere, in some cases at least 25 years ago. In contrast, the emergency contraceptive became prescription-free in Austria in 2009, six years before the usually less restrictive Germany, and approximately eight years after the United Kingdom (UK), and France [3, 9]. Research has explored reasons for such variability. The research has focused on the schedules of medicines [10, 11] and processes [12–15]. Recent, broader perspectives [16–18] suggest that factors affecting reclassification activity include government support, population size and market exclusivity. Also influential are pharmacy-only or pharmacist-only schedules, pharmacy interest and a self-medication culture. The regulator and committee perspective, medical support or opposition, the cost and effort of making applications, funding for prescription and prescription co-payments, and certain individuals have a further impact.

Austria’s health costs are above those of many other European countries and are expected to increase, raising concerns about the health system’s fiscal sustainability [19]. The Austrian health insurance fund expects a deficit in 2020 of €400 million with reduced company contributions resulting from unemployment related to the Covid-19 recession [2]. Reclassification might help. Although Austria is comparatively non-progressive [5] and industry in Austria has low interest in switch [15], the enablers and barriers to reclassification in Austria have not been investigated. Addressing this problem could indicate why its consumers have less access to medicines through reclassification than do consumers in many comparable countries. Therefore, this paper aims to elucidate forces shaping reclassification in Austria vis-à-vis other countries, and use this evidence to inform recommendations for change.

## Methods

This qualitative research builds on previous research by two of the authors. The University of Auckland Human Participants Ethics Committee approved this research (020041).

Study participants were purposively selected to vary maximally from the Prescription Committee, (“Rezeptpflichtkommission”), Ministry of Health; medicines regulatory agency, AGES; pharmacy, medical and patient organisations; health insurance; academia (pharmacy, health economics, and pharmaceutical policy analysis); industry; and politicians (Table 1). Following an approach by the lead author through email and/or telephone, and written informed consent, participants gave personal interviews with her face-to-face in English in June 2018. Exclusion criteria were unavailability for a face-to-face interview during the period of interviews and not providing written informed consent. Two politicians, a pharmacy academic and an industry person invited to participate were unavailable or did not agree to participate.

**Table 1:** Study participants.

	Number interviewed*	Number of interviews*
Committee members	7	6
Patient organisation representative	1	1
Pharmacy academic	1	1
Industry representatives	5	3
Pharmacy organisation representatives	6	3
Medical organisation representatives	2	2
Government employee from AGES or the Ministry of Health	5	4
Health insurance representative	2	1
Economist academic	1	1
Pharmaceutical policy analysis researcher	1	1
Politician	1	1
Academic pharmacologist	1	1

AGES: Österreichische Agentur für Gesundheit und Ernährungssicherheit GmbH.

*Participants could belong to multiple groups, e.g. committee member and government employee.

The lead researcher brought international experience in medicines reclassification research, policy and practice and a background in pharmacy practice. An “insider” member of Austria’s medicines regulator complemented both her “outsider” perspective and that of the third author (an internationally recognized academic in primary health care), as non-Austrians. Use of a heuristic study design [20] brought to the fore the professional and personal experiences and insights of these researchers. A scientific advisor to the project, Dr Christa Wirthumer-Hoche from the Austrian Medicines and Medical Devices Agency, part of the Österreichische Agentur für Gesundheit und Ernährungssicherheit GmbH (Austrian Agency for Health and Food Safety Ltd AGES), provided input into the list of proposed participants, interview guide and draft report.

The topic guide (Table 2) centred around barriers and enablers to reclassification, the reclassification process and possible improvements, and relevant aspects of the health system. We based this guide on previous research [17, 18]. Questions were tailored to each participant’s role and information arising during the interview (S1 Panel). Given the focus on maximum variation sampling, the intention was not to achieve saturation as a discrete event indicating no further need for data gathering, but to identify ‘new’ themes as part of a process of moving toward conceptual depth.

**Table 2:** Topics typically covered in the interviews*.

• Barriers and enablers to reclassification
• The process of reclassification
• Experiences of the processes and committee meetings
• Potential improvements to the process or changes to committee membership
• Application quality and possible improvements
• Views on reclassification generally
• The ability of pharmacy to manage the medicines
• Consumer culture around healthcare
• Access to doctors and the health system model in Austria
• Research on reclassifications
• Potential areas for reclassification

*Not all topics were covered in all interviews.

Interviews were audio-recorded and transcribed verbatim, then sent to participants who could modify them. Analysis used a general inductive approach, managed through Nvivo 11 software.

Interviews were read and re-read, then coded by the lead author using coding nodes relevant to the study aim based on areas of discussion. Similar and related codes were grouped into themes. Data were analysed systematically by each code ensuring reporting was comprehensive. Analysis focused on commonalities and differences across participant groups and the whole sample. Where requested, quotes were confirmed with participants before use.

Austrian findings were compared with data from Germany [18], a country similar to Austria in terms of health system [21] and health expenditure [22], and a multinational study [16, 17, 23] using the judgement of the author leading each study. Scientific advisors’ suggestions on the draft report were actioned.

## Results

Twenty interviews were conducted with 24 key informants (Table 1) for 30 to 100 minutes each with most taking 60 minutes. Box 1 outlines the process of reclassification and committee constitution outlined by participants and AGES documents.

**Box 1. Process of reclassification from interviews and documents.**

Typically applications come from pharmaceutical companies, but anyone can propose a reclassification, including the medicines regulator. Before submission, the sponsor can discuss the reclassification with AGES, the medicines regulator, in a scientific advisory meeting. A reclassification application is typically submitted to the Chamber of Commerce which seeks the opinion of companies with products containing that ingredient, before forwarding the application to AGES.

AGES evaluates the submission, considering the European Directorate for the Quality of Medicines and HealthCare (EDQM) recommendation. The Rezeptpflichtkommission (Prescription Committee) considers the submission. The committee covers human and veterinarian medicine classification, including new medicines for prescription status, licence restrictions (e.g. use in children) and prescription to non-prescription reclassifications or reversals.

This committee comprises eight voting members: an AGES employee; a pharmacology academic; one representative each from the Chambers of Pharmacists, Physicians, and Veterinarians; an expert representing pharmaceutical manufacturers; a health insurance representative; and the Chair who is an official of AGES or the Austrian Federal Office for Safety in Healthcare. Typically members are reappointed indefinitely with some members already there for 20 years.

Committee members receive the application and a brief AGES document including its opinion, and sometimes an AGES presentation. An applicant can request to send an expert. If the committee agrees, this person may present and be asked questions, then leaves before the discussion and voting. The reclassification criteria strongly focus on safety with benefit-risk assessment unmentioned. Sometimes the committee discusses the application before its members vote. A majority decides the committee recommendation which the Minister of Health can follow or not. This reclassification is by ingredient, not brand, and there is no market exclusivity, allowing fast generic entry.

Unusually, Austrian law also allows an alternative pathway involving a mutual recognition procedure with AGES, which considers the reclassification within that process unless it chooses to refer the application to the Prescription committee. This brand-specific reclassification reportedly benefits companies by delaying competitor generic entry. For example, racecadotril for diarrhoea (by brand) was reclassified through this process. A mutual recognition procedure approval without committee referral is more likely with medicines with non-prescription packaging, which are non-prescription in multiple countries.

### Reclassification inactivity

Many participants considered Austria to have fewer reclassified medicines than some other countries for two key reasons: few submitted applications, and committee rejection of applications. A bidirectional relationship emerged. Reportedly expecting negative committee opinions, companies had not submitted applications. With few applications, little familiarity with reclassification prompted committee caution. Two government participants noted the lack of applications:

*…it’s absolutely gone to zero*. *I don’t know why… On the other hand*, *here in the agency we have two to four [applications] a year as a variation procedure…*. *because the chances are higher*.

Having only one reclassification application in recent years limited the ability of committee members to comment on application quality, reclassification process or potential improvements to either. Moreover, the last application received little discussion before rejection:

*… it was a cortisone*, *cortisone was on prescription in Austria*, *and … this was not possible*, *this was our discussion about this topic*.

Likewise, industry lacked experience of preparing applications, selecting candidates, or the reclassification process. Strongly discouraging companies from submitting applications were the limited market associated with a small population, fewer products registered than elsewhere, little self-care and advertising restrictions on reimbursed reclassified products.

#### Culture

Culture emerged strongly as a factor affecting consumer behaviour, decision making, and stakeholder and politician behaviour. Several participants indicated that because the *“social state … looks after everything”*, consumers unnecessarily used the doctor or hospital, rather than taking *“initiative”* with self-care or becoming informed. Insufficient self-reliance (compared with higher levels in Anglo-Saxon countries) was suggested by an academic participant to reduce public and political interest in reclassification, and by a committee participant to affect how the committee viewed consumers, e.g. assuming they would not read labels. However, several participants suggested consumer empowerment and self-reliance were increasing.

Conservatism, risk-averseness and slow change commonly arose. Four participants believed that thalidomide (which had been prescription-only in Austria versus non-prescription in Germany) caused committee risk-averseness. However, a government participant considered this historical concern was irrelevant now within a robust regulatory environment. Meanwhile, participants suggested that change-aversiveness or expecting policy change to be slow characterized the committee and society generally:

*…things take 5–10 years to come from Germany to Austria… when we’ve seen it’s okay in other countries then we might adopt the procedure*. *Politician participant*

Various participants reported reclassifications being rejected to avoid a cascade of other changes (quote below). Others described an acceptance of processes that *“do not make much sense”*. The Minister of Health typically accepted the committee’s recommendation even when it differed from other countries’ decisions (except for the emergency contraceptive). For example, dermal hydrocortisone long reclassified elsewhere was thought impossible in Austria:

*Nobody would ever say hydrocortisone is a problem*, *but everyone would say no we do not have cortisones in Austria*. *It would open up doors for others and it’s written in the law that all cortisones must be [prescription]*. *Government participant*

A culture of co-operation and conflict avoidance commonly arose, with three participants specifically mentioning that *‘Social Partnership’* (Sozialpartnerschaft) hindered reclassification. Social Partnership backs representative committees rather than expert committees with a culture that attempts to reach consensus and avoid conflict, which hindered reclassification. The prescription committee represented mainly government and interest groups, rather than being an expert committee, and had no consumer voice. The committee attempts to reach consensus, and the Chamber of Commerce seeks agreement across product sponsors before forwarding a reclassification application to AGES (Box 1). A significant change, such as vaccinations in pharmacy, would reportedly first require discussion with politicians and different groups, and gaining agreement could take time and require trade-offs.

#### Politics and medical power

Despite concerns about increasing health spending, participants suggested reclassification was *“not on the agenda”* politically–consumers, patient groups and media reportedly had low interest, and doctors had powerful lobbies. However, politics enabled the 2009 emergency contraceptive (levonorgestrel) reclassification. The Minister of Health prompted the sponsor to submit an application. Then, despite a negative committee recommendation, the Minister uniquely reclassified it, with participants suggesting that this action was motivated by an upcoming election or women’s rights and the political environment, frustrating some committee participants.

The two medical participants considered that accessible doctors minimised the need for reclassification, while acknowledging that self-care was sometimes appropriate. Conversely, four participants reported that, without an appointment system, consumers could wait up to three hours for doctors sometimes, and that some shortages of speciality and rural doctors existed.

Some non-doctors suggested medical opposition reflected fear of losing earnings and power. Doctors could ill-afford reclassification because GP consultations were brief (*“2–3 minutes”*) with low income generation (€25/consultation), and funding limits on frequent visits:

*… the business model relies also on people which you just need a couple [of] minutes because it’s such a simple case*. *Academic health economist*

Five participants suggested that the Hausapotheke (doctors’ dispensary) was an important income source for doctors and used instead of a pharmacy, impeding self-medication. Participants reported medical influence through the committee, politics, and the media. Additionally, pharmacy did not want to offend doctors.

Participants from academia, politics, industry, pharmacy and the patient’s organisation volunteered that medical-political strength significantly hindered reclassification, needing their agreement for change. Demonstrations or strikes by doctors (supported by media) might happen, “*and then you lose the political debate*.*”*

*[Doctors] were able to stop certain reforms*, *or to slow them down so… the reason [progress is limited] has to do with doctors … Academic participant*

### Pharmacy issues

Pharmacy issues commonly arose, including sub-themes related to pharmacy’s interest in reclassification, pharmacy’s standing, trust in pharmacy, access, education, and competition. Academics, a patient representative, the politician, and insurance and pharmacy participants suggested pharmacy could take a more significant role:

*… it would make sense to include pharmacists in an increased manner for simple services such as vaccinations or self-medication…*, *for monitoring blood pressure*, …. *improving system efficiency and … accessibility… Health economist*

Some participants proposed that reclassification would raise pharmacy’s profile or status. However, a medical participant reported some distrust of pharmacists by doctors. Participants including committee and government mentioned that negative mystery shopping findings, e.g. selling antibiotics without a prescription, eroded trust in pharmacy:

*If we think about job enrichment and doing shots [vaccination] and things that doctors do*, *do your job first! There’s a little gap between what they should do and what they really do*. *Health insurance participant*

Conversely, *“quite positive”* orlistat post-reclassification mystery shopping findings were also reported. Nevertheless, concerns were expressed about minimal undergraduate teaching on providing non-prescription medicines. Some participants wanted undergraduate education to include pharmacy practice to better prepare pharmacists, for example on *“how to speak to a patient*.*”* A new pharmacy school teaching clinical practice was welcomed because it could increase clinical skills and pharmacy practice research. Pharmacy organisations provided training and consulting guides following reclassifications.

Some participants observed that legally pharmacists could not diagnose health problems, and a committee member worried that *“the pharmacist is not responsible for his treatment with an OTC product”*. Although accessibility to pharmacy and reduced waiting times were often mentioned, some participants noted limited pharmacy hours, e.g. closing noon Saturday, and no pharmacies in Hausapotheke (doctor dispensary) areas. A medical participant worried about pharmacists’ advice and patient safety, and wanted reclassification limited to minor ailments previously experienced, but appeared unconcerned about internet supply of non-prescription medicines. Another was as comfortable with drugstore supply (with no pharmacist) as with pharmacy supply, providing both gave advice. One medical participant preferred greater use of nurses rather than expanding pharmacist activities, and the other disagreed with messages for patients to *“go first to the pharmacy”*.

While some participants suggested pharmacy was strong, others suggested 40,000 doctors were stronger than 1,400 pharmacies. Until recently, participants noted that pharmacy has mostly given only limited support to reclassification. Concerns included wanting to avoid offending doctors, loss of earnings from internet sales (permitted for non-prescription but not prescription medicines), further reclassifications to the drugstore, and low return for some non-prescription medicines. With 70% of business reportedly from prescription dispensing, it is unsurprising three participants noted pharmacists wanted to avoid offending the doctors:

*We don’t want to interfere*, *[the doctors] are our partners and we don’t want to attack or take something away*, *we want to help them*. *Pharmacy participant*

The *‘Notfall’* [emergency] rule, allowing pharmacists to provide prescription medicines in an emergency, possibly reduced the need for reclassification. Participants reported that the pharmacist evaluated emergency status, based on criteria such as *“the availability of a general practitioner*, *the urgency of dispensing and the type of medicine”*. Inconsistency was evident, e.g., some participants believed that a pharmacist could not supply an antibiotic for cystitis, while others indicated that *“in real-life she would get the medicine”*, perhaps with a prescription afterwards. Reporting emergency supply was sometimes used inappropriately for medicines that were non-prescription in other countries, e.g. nasal corticosteroids, a pharmacy participant suggested reclassification would be *“a legalisation”*, and a government participant observed that the emergency rule was not ideal because of inconsistencies in its application.

The interviews conveyed a feeling that the pharmacy mindset was moving towards more clinical work, new services, a higher profile, and therefore reclassification, sometimes influenced by Switzerland and Germany, and aiming for highest level practice, and benefit *“especially for the patient”*. Potential challenges of reduced dispensing funding and drugstore encroachment were suggested to aid the development of such an interest.

#### Financial effects

Financial effects wove throughout the other themes. Examples included consumers preferring subsidised health care to funding self-care, doctors’ reported need for many short consultations given the payment model and doctors’ dispensaries, limited market attractiveness for pharmaceutical companies, and pharmacists worrying about post-reclassification revenue loss to internet sales.

### Aspects of the committee and process

Various participants suggested the committee was conservative (see also Culture), in recommending rejecting medicines reclassified elsewhere, e.g. nasal corticosteroids and emergency contraception. Some suggested the committee’s conservatism was aided by a medical bias which was called an *“imbalance”* by one government participant who indicated that three of the seven-member committee typically reflected a medical position.

However, a government participant considered two recent rejections were bad luck: nasal corticosteroids affected by the law that corticosteroids are prescription-only; and low dose diclofenac affected by negative findings published just before the committee meeting.

Some committee members’ engagement with reclassification seemed limited:

*I just go there*, *I do my thing and then I accept what I have heard because I know that eventually it can be overridden*, *and … the Rezeptflichtkommission [is not] as important as the reimbursement committee*.

*… it is not really a very*, *very sexy thing*,… *someone comes late …*, *“sorry I’m late*, *what’s going on?” “Oh this and this.” “Okay I’m against it*.*”*

Similarly recall was sometimes also impaired. Three committee members erroneously thought that statins or mifepristone had been considered and another committee member stated sildenafil had been reclassified. The regulator confirmed that the committee had not considered these medicines. Two participants suggested that some members relied on the AGES document and their own opinions of the medicine rather than considering the application. Some committee members opined that the discussion was sometimes insufficient and/or that some members had decided in advance of the meeting, sometimes following their organisation’s preference. Nasal corticosteroids were rejected (given the law mentioned above), reportedly without discussion of risks or benefits. Conversely, one participant described the emergency contraceptive discussion as *“robust”* before the negative recommendation.

Industry and government participants supported the dual reclassification process (committee or registration pathway), with an industry participant observing that without the registration option no reclassifications would happen. However, anything contentious (even topical hydrocortisone long reclassified elsewhere) was referred to the committee. Industry was frustrated by an opaque process with no publicly available agenda, or minutes, which meant it was unknown which medicines had been considered, and no justification was released for decisions. Thus, industry wanted greater transparency and a process outline.

Several industry and pharmacy participants appreciated the openness of AGES to reclassification. A committee member reported that the AGES report often favoured the reclassification, and AGES drove a reclassification for flurbiprofen lozenges. Government participants wanted more applications: *“there is so much room for improvement and possibilities to strengthen the market for self-medication*.*”*

No one volunteered that market exclusivity was needed, but a government participant saw market exclusivity requiring post-reclassification research as enabling, provided companies followed through.

*… let’s make a conditional switch*. *Accompany with a trial to see if it’s working*. *I think it’s a very good idea … we base it really on a scientific basis*.

While most committee members were happy with the amount of data provided, one committee member wanted less and government participants wanted application improvements, including more data.

*They just say we want this as an OTC product and we say “okay bring us some data”*. *“We do not have data*, *but in Germany it’s OTC*.*” “You have to give us pharmacovigilance data or something”*. *But the data quality is… to my impression is not that good at the moment*.

Some participants suggested more experts and a (non-medical) patient representative on the committee would improve balance.

### Comparison with other countries in major barriers and enablers

Valuable as these findings are, they become more useful when compared with other high-income countries which have been studied in a similar way (Germany in 2017, and New Zealand, Australia, UK, US and Japan in 2009–2012) [17, 18]. Austria generally had more barriers and fewer enablers than all other countries, and this is shown for Austria and Germany (Table 3).

**Table 3:** Comparison of critical enablers and barriers to reclassification across Austria and Germany*.

	Austria	Germany
Self-medication culture	+/-	+++/-
Population and market size	--	+++
Medicine schedules		++
Advertising of non-prescription medicines	+/-	++/-
Individuals	+	+
Politics and government support	-	-
Prescription co-payments	--	-
Medical access	--	+/-
Loss of medicine reimbursement if reclassified		--
Pharmacy organisation involvement	+/-	+/-
Medical support/opposition	--	--
Pharmaceutical industry environment	++	+++
Industry confidence in committee	--	+
Working with the regulator	++	++
Market exclusivity		-
Regulator/committee openness to reclassification	+/--	+/-
Patch protection	--	--

*Barriers are represented by–, enablers by +, and mixed factors by +/-; quantity represents strength of effect. Where a factor was discussed, but appeared to have no effect, 0 represents the effect. Where a cell is blank, the factor did not arise. This chart is subjective, according to how participants have communicated the factors, and the lead researcher’s interpretation. It is not exhaustive. Interviews were conducted in Austria in 2018 and in Germany in 2017.

Austria differed significantly from its neighbour Germany, particularly in population, and cultural aspects, e.g. minimal self-care and the consensus-driven nature of Austrian society, inhibiting reclassification. Most German committee participants strongly desired more data in applications, while their Austrian counterparts generally did not. However, both countries had some regulator openness to reclassification, concerns about committee composition and process, medical opposition to reclassification, insufficient pharmacy undergraduate clinical training, lack of pharmacy practice research, pharmacy organisation ambivalence and committee concerns about pharmacy and consumer capability. Austrian committee participants were generally more positive about a patient representative on the committee than German committee participants.

Austria and Japan shared a culture of visiting a doctor, conflict-avoidance, and medical power limiting reclassification. While the large population size was strongly enabling in some countries (Germany and the US particularly), the small population and small market size hindered reclassification in Austria. However, NZ had a similarly small population and market size, clearly inhibiting reclassification, but NZ was comfortable leading reclassification. Its committee was flexible and open to reclassification, and change was common and accepted culturally, making NZ progressive despite being an *‘unimportant’* market. Conversely Austria preferred a follower position, with a culture that expected and accepted minimal or slow change, and the committee appeared inflexible and conservative. The committee inflexibility and conservatism in Austria had some similarities to Australia at the time of the earlier research. However, lack of applications and committee engagement did not emerge in Australia. Market exclusivity was a strong enabler in Japan and the US, but did not appear in Austria, while lack of market exclusivity in Germany emerged as a barrier.

## Discussion

Consumer access to non-prescription medicines is more limited in Austria than many developed countries [5], with potential effects on the health system, funding and consumer engagement in their health care. This research found many barriers to reclassification and a few enablers in Austria. Barriers included a: small market, conservative committee, powerful medical lobby, culture of change-averseness, preference for consensus, and limited self-care. Enablers included the dual reclassification process and regulator openness to reclassification.

While there are several commonalities with other countries, some findings differed substantially in Austria from other countries, in part because reclassifications and applications were uncommon. Important factors elsewhere of company capability for reclassification, and market exclusivity (delaying competitor entry post-reclassification) [23], were unvoiced in Austria, probably because larger barriers discouraged companies. Having few applications affected committee members’ ability to comment knowledgeably on applications, and likely also affected their engagement on the committee, which was, understandably, seen as unimportant. Others have also noted low industry interest in switch in Austria [15].

The cultural influence of a consensus-driven environment with a social partnership, and acceptance of slow change, differed from other countries examined. Others have reported difficulties of Austrian political culture in starting a rational public discussion about optimising primary care [24] or delaying change in assisted reproductive technology regulation [25]. The latter research also commented on the social partnership, with policy-makers being an exclusive group with low turnover and having a paternalistic relationship with the public whose citizens seldom participate in policy-making. Similarly, the classification committee members were also often long-standing, included no consumer voice, and reportedly did not entirely trust the public and pharmacy or use a benefit-risk approach. Furthermore, there was a lack of transparency and no public consultation. The lack of consumer voice at the committee and lack of public consultation is surprising given a long-standing intent in Europe to have public participation in health decision-making, and an increasingly people-centred approach [26]. However, most other European countries also have no consumer voice and no public consultation in reclassification of medicines [15]. Given that better outcomes are expected from patient and public involvement in pharmaceutical regulation, including increased transparency and trust and higher quality scientific committee opinions [26], Austria and most European countries could address this area. A transparent risk-benefit approach is likely to aid decision-making for reclassification [27].

Important but also unusual was pharmacists’ ability to supply some medicines in emergencies without a prescription, reducing the need for reclassification. Similar findings arose in the US where collaborative prescribing and other state-based initiatives have opened access to some medicines [17]. However, such supply has no manufacturer-provided consumer labelling and (in Austria) no additional training of pharmacists in that therapeutic area or tools for supply. Moreover, availability may vary between pharmacies. Thus, a reclassification may sometimes be preferable to widen appropriate consumer access to medicines.

The culture of consensus and conservatism seemed so strong that Austria is unlikely to become a reclassification leader. The committee appeared conservative in recommending rejection of reclassifications long approved in some other countries [3], e.g. the Austrian committee rejected the emergency contraceptive. In contrast, the German committee recommended approval, acknowledging the considerable evidence for this reclassification [18]. However, Austria could become more progressive and, since this research, has reclassified topical hydrocortisone up to 0.5% [28]. Transparent decision-making that is more clearly evidence-based would raise confidence in the committee. Meeting minutes including justifications for decisions should be published, as in many other countries. The committee could usefully include a patient representative to hear the patient voice, and a pharmacy practice expert, and have limited terms for members. High quality applications and local pharmacy practice research would inform committee considerations. Government support for reclassification, as has occurred elsewhere [17, 29], would be enabling.

Insufficient self-medication options might discourage self-care, and Austrians have low rates of self-care [30], despite some reports by consumers of lack of time to attend doctors, and some frustration with health check visits [31]. Reclassifying more effective medicines might encourage consumer self-reliance and political interest, particularly given reportedly long waits in waiting rooms for doctors in our research, and increasing health care costs [19], burden on hospital outpatients services, and relatively low numbers of general practitioners [19]. However, the underlying culture may take time to change.

We found significant differences between two similar neighbouring countries: Germany and Austria. A previous comparison between Australia and New Zealand [16] also found important differences between similar neighbouring countries, particularly in committee conservatism in Australia versus openness in New Zealand, but was limited by the small number of committee members interviewed. This current research study gave greater insights into committee aspects both within Austria and with the neighbouring comparator Germany because of the larger number of participants including many committee members.

### Strengths and limitations

Many key informants were interviewed, including those closely involved in reclassification and important stakeholders, e.g. a politician and patient representative. The previous international work [16, 17] aimed for breadth rather than depth, while this research with 24 participants in a single country provided considerable depth.

The mixed “insider-outsider” approach facilitated the data collection and helped to manage potential recall errors from participants. However, it opened the researchers to bias, which they sought to address reflexively through seeking a range of perspectives, inviting participants to review their transcripts, using systematic analysis, and using skeptical peer review.

### International implications and future research

Different barriers and enablers to reclassification have been identified for Austria and compared across other countries. Such comparative research across national and sociocultural boundaries offers an opportunity to learn from the different experiences and approaches taken by these health systems in the context of increasing globalization. Lessons from Austria include the findings that reclassification requires a progressive culture including political support and a market large enough to encourage applications from companies that can expect transparent, evidence-based decisions that account for benefits and risks.

With a relatively small population, small potential market and a committee perceived as conservative, industry was not focused on reclassification in Austria. Similarly, Sweden has a small population size, and has been considered conservative [32]. However, Sweden reclassified omeprazole in 1999 (government-driven) [32], and triptans in 2007 [33]. In 2009 it widened access to many medicines via non-pharmacy outlets [34], and thus appears more progressive than Austria. A small population emerged as an important barrier to reclassification in Denmark, New Zealand, Australia and Singapore in earlier research, but flexibility and proactivity helped overcome this [17]. For example, the medicines regulator or committee have suggested reclassification candidates and/or the government has progressed widened access to medicines without a company application in Denmark [35], New Zealand [36], Singapore [17], Sweden [32] and, more recently, Ireland [37, 38].

The variable support from pharmacy which reflected the emergency supply allowances, and the potential for internet supply without prescription, were similar to pharmacy ambivalence in some other countries [17, 18]. Research on Austrian community pharmacists’ views on reclassification, as has occurred recently elsewhere [39–41], would reveal whether the pharmacy perspectives captured in the research were representative or not of the members of the profession.

This research found the committee composition, engagement by individual committee members, and the process and quality of applications to be fundamentally important. Changes in committee composition, process and applications were similarly recommended for Germany [18], and the importance of individual committee members arose in Australasia [16]. Brass and Hiatt [42] suggested improvements to US Food and Drug Administration (FDA) committees, including improved preparation for meetings; mandatory committee member training; enhanced committee member engagement and openness; changes in committee composition; and more informed committee discussion. Functionally and geographically isolated, reclassification committees could benefit from cross-pollination, e.g. through Chairs meeting regularly to share information of international relevance to reclassification. Research is needed into the optimal committee composition and processes for reclassification and how to maximise the quality of deliberations.

## Conclusions

This research has highlighted the extent of variation between countries in medicines reclassification. New findings emerged from Austria including the enabling, dual process of reclassification in Austria, hindrance of the ‘*Sozialpartnerschaft’*, and expectation of slow or minimal change. Other barriers such as conservatism, medical opposition, low consumer self-care and limited market size seen elsewhere are particularly strong in Austria. The research reinforces the importance of the committee composition, reclassification process and culture of low self-sufficiency. Encouraging reclassification by addressing some barriers could provide more consistent availability of medicines to consumers (rather than relying on emergency supply), similar to other developed countries, help health funders manage increasing costs and reduce waiting times. These benefits may be particularly important in the current economic environment. For Austria, committee composition and process changes, and government support for self-care, would probably most enable further self-care options.

## Supporting information

S1 Panel Question guide for interviews. (DOCX)
