# Supplementary material for: Why are self-medication opportunities limited in Austria? An interview study and comparison with other countries
Source: PLoS One. 2021 Jan 25;16(1):e0245504. doi: 10.1371/journal.pone.0245504 (PMC7833129; doi:10.1371/journal.pone.0245504)
Supplement: S1 Panel — (DOCX) [file pone.0245504.s001.docx]

**S1 Panel.**

**Question guide for interviews**

Note: this is a guide only. Interviews were semi-structured, the actual questions asked and order of questions differed according to the conversation and the role and experience of the person being interviewed.

1. Describe your/your organisation’s role in the reclassification of medicines from prescription to non-prescription.
2. Can you tell me what you think about medicines being available without a prescription in Austria?
3. What do you think about moving medicines from prescription-only to being able to buy them at a pharmacy?
4. Who do you think benefits from this?
5. What are the risks?
6. What effect does this reclassification have on …… [e.g. doctors, consumers, pharmacy, according to participant]
7. Where do you think Austria sits internationally or across Europe in the range of medicines available without a prescription?
8. Why do you think that is?
9. What do you think are barriers to reclassification happening in Austria? [can probe on specific areas as needed, e.g. committee, process, scheduling, industry, plus specific questions below]
10. What do you think might encourage reclassification to happen in Austria? [can probe on specific areas as needed, e.g. enabling aspects to the committee or process, scheduling, industry, plus specific questions below]
11. What about pharmacy – how does that influence reclassifying medicines from prescription to non-prescription?
12. How about the general health environment in Austria, is there anything there that helps or prevents reclassifications from happening? [probe why as necessary, consider health funding, role of doctors, accessibility to doctors]
13. How about the culture of the people with medicines, is there anything there that helps or prevents reclassifications from happening? [probe why as necessary]

Questions for those are aware of the process of reclassification choose from the following questions or similar, concentrating on their areas of expertise, e.g. applicant, committee member.

1. How about the process, what aspects of the process help reclassification?
2. What aspects of the process slow down or prevent reclassifications?
3. What improvements (if any) would you like to see to the reclassification process?
4. Is there anything about the committee process that helps reclassification? [if so what?]
5. Is there anything about the committee process that you think stops reclassifications happening? [if so what?]
6. If not covered probe as is relevant to the participant: the committee (e.g. committee meeting generally, committee membership, applications provided e.g. quality, size, information included, ability to have an expert for the company present, evaluation), the process
7. Are there any changes that you would like to see happen for the committee membership?
8. Are there any changes that you would like to see happen for the committee process?
9. Within the committee meeting, is there anything that helps reclassification?
10. Within the committee meeting, is there anything that hinders reclassification?
11. As a committee member, how useful to you are the applications from the companies? [probe if necessary on quality, volume, readability, need for more evidence from studies.]
12. What improvements could you recommend, if any?
13. How does the evaluation affect the reclassification? [could probe on the interest as in Australia to have the evaluation go to the applicant for comment before going to the committee]
14. Companies say they need to have market exclusivity for more than one year to make a reclassification financially viable. What do you think?
15. Japan allows 3 years market exclusivity if post-marketing surveillance is conducted. The US provides 3 years market exclusivity if pre-reclassification trials are conducted. What is your view on these options?
16. Are there any factors that affect reclassification that we have not discussed?
17. Of all of the things we have discussed, what do you think is the most important?
18. What is the number one change you would like to see happen?
19. Do you have any final comments?
